# Efficacy and safety of *Curcuma longa* essential oil to inactivate hydatid cyst protoscoleces

**DOI:** 10.1186/s12906-019-2527-3

**Published:** 2019-07-26

**Authors:** Hossein Mahmoudvand, Mahbobeh Pakravanan, Mohammad Reza Aflatoonian, Amal Khudair Khalaf, Massumeh Niazi, Seyyed Reza Mirbadie, Amir Tavakoli Kareshk, Mehrdad Khatami

**Affiliations:** 1 Razi Herbal Medicines Research Center, Lorestan University of Medical Sciences, Khorramabad, Iran; 2 Central Research Laboratory, Deputy of Research, Kerman University of Medical Sciences, Kerman, Iran; 3 Research Center for Tropical and Infectious Diseases, Kerman University of Medical Sciences, Kerman, Iran; 4 Department of Microbiology, College of Medicine, Thiqar University, Nasiriyah, Thiqar Iraq; 5 Student Research Committee, Lorestan University of Medical Sciences, Khorramabad, Iran; 6 School of Medicine, Shahroud University of Medical Sciences, Shahroud, Iran; 7 Infectious Diseases Research Center, Birjand University of Medical Sciences, Birjand, Iran; 8 NanoBioElectrochemistry Research Center, Bam University of Medical Sciences, Bam, Iran

**Keywords:** GC/MS, Cystic echinococcosis, *Echinococcus granulosus*, Protoscoleces, Turmeric

## Abstract

**Background:**

One of the most important ways to cure hydatid cysts is surgery. Today, available chemical drugs have shown severe complications during hydatid cyst surgery. Here we investigated theefficacy and safety of *Curcuma longa* (*C. longa*) essential oil (CLEO) against hydatid cysts protoscoleces.

**Methods:**

Here, the collected protoscoleces from fertile hydatid cysts were exposed to different concentrations of the CLEO (50–200 μl/mL) for 5–30 min in vitro and ex vivo. In addition, male NIH mice (48 mice) were applied to examine the safety of CLEO.

**Results:**

All protoscoleces were completely killed in 5th min of treatment to the doses of 200 μl /mL of CLEO. On the other hand, in the 10th min of treatment, CLEO entirely killed 100% of protoscoleces at the dose of 100 μL/mL. Other doses of CLEO, but, revealed a postponed activity. Although CLEO at the doses of 50, and 100 μL/mL exhibited no similar effect in the ex vivo analysis; but, at the dose of 200 μL/mL and an exposure time of 5 min, approximately 100% of protoscoleces were destroyed into the cyst. We found that after intra-peritoneal injection of the CLEO for 14 days, although blood and biochemical parameters were changed; but there was no significant difference in comparison with the control group (*p* > 0.05).

**Conclusion:**

This research demonstrated that *C. longa* revealed the promising scolicidal effects against protoscoleces in vitro and in vivo, of course, after additional tests; it might be considered as an herbal scolicidal drug in order to decrease the threat of discharge of protoscoleces through hydatid cyst surgery. Nevertheless, supplementary studies will be desired to prove the current findings by examination the essential oil in a clinical setting.

## Background

Cystic echinococcosis (CE), as well known as the echinococcal disease is an infection occurred by *Echinococcus granulosus* which is observed in the small intestine dogs [1]. CE has been documented as a significant monetary and community well-being worry in various parts of the word particularly in developing countries [2, 3]. Humans along with domestic livestock can be the intermediary hosts in whose various organs it creates hydatid cysts [2, 3]. Because cysts are gentle-developing, infections with CE cannot create any symptoms for many years. However, the burst of cyst fluid may result in allergic responses or even death [3]. Currently, clinical managements for CE are based on surgery, percutaneous methods and chemotherapy for live cysts, as well as “watch and wait” method for silent cysts [4]. Likewise, chemotherapy with benzimidazoles has been applied to take care of hydatid cysts and demonstrated effectiveness against CE. However, they represented a range of complications such as hepatotoxicity and alopecia [4, 5]. Up to now, surgery is the favored treatment for CE, however, it has threats including those of surgical involvement, anaphylactic shocks, and secondary CE because of spilling of the contents of cyst which called protoscoleces [5, 6]. To date, available chemical drugs including 20% hypertonic saline, Ag-nitrate, and cetrimide have been applied in surgical and percutaneous methods, but they have shown severe complications for example biliary tract fibrosis, necrosis of the liver, and methemoglobinemia [7, 8]. Thus, it is compulsory to discover a perfects scolicidal agent with high effectiveness and no local or systemic complications for hydatid cyst surgery.

The use of herbs and spices has a very long history in folk medication to cure of various diseases and illnesses. Nowadays, herbal medicines supply boundless chances for discovering novel agents for therapy an extensive range of diseases as a result of possessing few complications, a low cost, and high accessibility [9]. Spices have been applied for centuries to get better the flavor and aroma of foods as well as for their medicinal properties. Spices are also famous for their antimicrobial features and are the most frequently used natural antimicrobial agents in foods [10]. In modern medicine, essential oils from various spices have been found to possess different antimicrobial effects, and the majority of them are categorized as ‘Commonly Recognized as Safe’ [11].

*Curcuma longa* L. from the family of Zingiberaceae with the familiar name of “turmeric” is extensively cultivated in tropical parts of Asia and Africa [11]. The plant possesses various biological activities such as anti-inflammatory, hepatoprotective, antimicrobial, wound healing, anticancer, antitumor, and antiviral ones [12–14]. Previous investigations exhibited the chemical composition of *C. longa* essential oil (CLEO) in detail [15, 16].; however, this composition is depended on a number of factors such as geographical origin, collecting period, and environment situations are able to affect the chemical composition and bioactivity of the herbs [17]. Based on what was said we decided to evaluate the composition of CLEO, its scolicidal activity on hydatid cyst protoscoleces in vitro and ex vivo, and its safety in animal.

## Methods

### Plant materials

The plants materials (rhizome) were prepared from a market in Kerman province, south-east of Iran. After plant identification by a botanist, a voucher sample of the plant was placed at the Herbarium of Pharmaceutics Research Center, Kerman, Iran.

### Isolation of essential oil

About 200 g of plant materials that were dried and powdered were used for isolation of essential oil through hydro-distillation method by means of a Clevenger-type device. Finally, the obtained EO was dehydrated over anhydrous sodium sulfate, and deposited in the refrigerator up to examinations [18].

### Gas chromatography/mass spectrometry (GC/MS) analysis of essential oil

Here, to perform the GC analysis we used a Hewlett-Packard 6890 with a HP-5MS column (30 m × 0.25 mm, film thickness 0.25 mm). To calculate the composition percentage we used electronic integration of FID peak zones with no use of answer factors alteration. Then linear retention indices for obtained components were evaluated by coinjection of the samples with a solution comprising homologous series of C8–C22 *n*-alkanes. In next step, GC/MS analysis was carriedout using a Thermoquest- Finnigan gas chromatograph equipped with fused silica capillary DB-5 column (30 m × 0.25 mm, film thickness 0.25 mm) coupled with a TRACE mass (Manchester, UK). To identify the EO constituents we used the evaluation their relative retention time and mass spectra in comparison with the standards Wiley 2001 library data of the GC/MS system or literature information [19].

### Collection of protoscoleces and viability

Protoscoleces were obtained from the liver of sheep infected with hydatid cyst which slaughtered at Khorramabad abattoir, Iran. The preparation of protoscoleces was performed based on the methods described elsewhere [6].; whereas the number of protoscoleces was adjusted to 2 × 10^3^ protoscoleces with more than 90% viability.

### In vitro protoscolicidal activity

In vitro protoscolicidal effects of CLEO at the doses 50, 100, and 200 μL/mL for 5, 10, 20 and 30 min was examined according to the method described elsewhere. After the protoscoleces was treated to the various doses of CLEO at the desired time, in the next step, the mortality rate of the parasites was studied by eosin test. Briefly, 50 μL of 0.1% eosin stain (Sigma-Aldrich, St. Louis, MO, USA) was added to the treated protoscoleces and then smeared on a glass slide, covered with a coverslip, and tested under a light microscope. Lastly, the mortality rate of percentages was calculated by counting killed protoscoleces in 300 protoscoleces [20]. In this test, live protoscoleces are colorless and displayed specific muscular motions and flame cell action, but due to penetration of eosin in the dead protoscoleces made it red. Normal saline containing Tween 20 and Ag-nitrate were also considered as a negative and positive control, respectively.

### Ex vivo protoscolicidal activity

In this study to evaluate the ex vivo protoscolicidal activity of *C. longa* essential oil, liver fertile hydatid cysts acquired from naturally infected sheep and goats were used. Firstly, more than 50% of the content of the cyst was aspirated to determine the viability of protoscoleces by eosin test. For each dose of *C. longa* essential oil (50, 100, and 200 μL/mL) three hydatid cysts were applied. *C. longa* essential oil was injected to cyst. Then some of the cyst fluid along with protoscoleces was aspirated at 5, 10, 20, and 30 min and in the next step, 0.1% eosin was placed to the precipitate. Finally, the mortality rate of protoscoleces was calculated by eosin test similar to in vitro assay [21].

### Toxicity effects

#### Animals

A total of 48 male NIH mice (6–8 weeks old) were used to this study; whereas mice were kept in a colony room with a 12:12-h light/dark cycle at room temperature. It should be mentioned that handling of mice was based on the standard rules for working with laboratory animals.

#### Acute toxicity

In the present study, different doses of *C. longa* essential oil (1–4 mL/kg) were injected intraperitoneally into four groups of six mice each to assess acute toxicity. The mortality rate of mice was calculated 24 h after the administration. Moreover, using the Probit test in SPSS software we calculated the LD_50_ values for this EO [22].

#### Determination of clinical chemistry and hematological parameters

In this investigation, the sub-acute toxicity of CLEO and subsequently evaluation of the biochemical and hematological factors was performed based on the method explained by Shakibaei et al. (2013). In summary, four groups of mice (each group contain 6 mice) were intraperitoneally received normal saline, CLEO at the doses of 0.15, 0.3, and 0.6 mL/kg, respectively, for 14 consecutive days. Following the experimental period, mice in all groups were anesthetized using Ketamine (100 mg/kg) – Xylazine (10 mg/kg); then abdomen was opened, and blood samples were collected from the heart, in the next step, sodium pentobarbital (70 mg/kg, i.p.) was applied as euthanasia drug. For hematological evaluation, some collected blood put into tubes with ethylenediaminetetraacetic acid (EDTA) anticoagulant, and subsequently a number of hematological factors such as hemoglobin (Hb), hematocrit (Hct), white blood cell counts (WBC), red blood cell (RBC), and platelet (Plt) counts were calculated.

In order to assess the biochemical parameters, after separating the serum from the remaining blood by centrifugation at 2000 *g* for 10 min, a number of clinical chemistry factors including aspartate aminotransferase (AST), alanine aminotransferase (ALT), alkaline phosphatase (ALP), creatinine (Cr), blood urea nitrogen (BUN), and bilirubin (direct and total) were measured by means of commercial diagnostics kits (Roche, Germany) [23].

### Statistical analysis

In vitro and in vivo experiments were carried out in triplicate. The results of this study were analyzed by SPSS software (SPSS Inc., Chicago, IL, USA). One Way ANOVA as well as *t*-test were utilized to assess the variations among tested groups. *P* < 0.05 was considered statistically significant.

## Results

### GC/MS analysis

As shown in Table 1, 24 constituents were determined which made up nearly 95.5% of CLEO. The key constituents were α-turmerone (27.1%), β- turmerone (21.8%), l-phellandrene (8.8%), and ρ-cymene (5.4%), respectively.

**Table 1:** GC/MS analysis of *C. longa* essential oil

No	Components	KI^a^	% Composition
1	α- Thujene	7.434	0.7
2	α- Pinene	7.66	0.5
3	β- Myrcene	9.48	0.8
4	l-Phellandrene	9.97	8.8
5	ρ -Cymene	10.47	5.4
6	1,8-Cineole	10.72	3.7
7	Limonene	10.76	0.5
8	α- Terpineolene	12.68	0.8
9	trans-Caryophyllene	22.43	1.8
10	α-Caryophyllene	23.25	0.5
11	α-Curcumene	23.84	4.4
12	α-Zingibirene	24.25	3.2
13	β-Bisabolene	24.58	0.9
14	β-Sesquiphellandrene	24.94	4.2
15	2-Phenyl-1-D1-Aziridine	25.87	1.47
16	β- Caryophyllene	26.12	0.9
17	β-Atlantone	26.25	0.9
18	1,3,5-Cycloheptatriene	26.35	1.4
19	Pyrazine	26.45	0.7
20	β- Bisabolene	26.87	0.8
21	γ-Curcumene	27.23	2.1
22	β- Tumerone	27.90	21.8
23	Ar-Tumerone	28.10	14.7
24	α-Tumerone	28.74	12.4
25	3-Fluorophenyl isocyanate	29.91	0.5
	α-Atlantone	30.20	1.6
	Total		95.5

^a^Kovats index on non-polar DB-5 ms column in reference to n-alkanes

### In vitro protoscolicidal activity

**Fig. 1:**
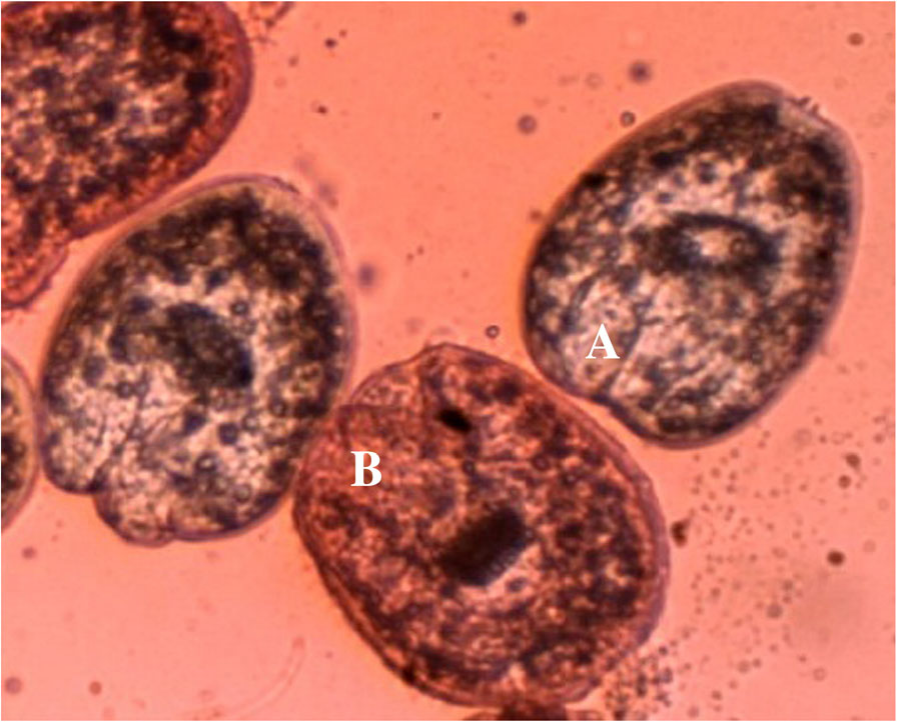
Live (**a**) and death (**b**) protoscoleces of hydatid cysts after exposure with 0.1% eosin

Table 2 shows the in vitro scolicidal effects of different doses of CLEO. The results demonstrated that *C. longa* essential oil displayed considerable protoscolicidal activity in all concentrations when compared with the control group (*P* < 0.05) (Fig. 1). The mortality rate of protoscoleces was 100% after 5 min of treatment to the concentration of 200 μL/mL of CLEO. Moreover, after 10 min of treatment, the protoscolicidal activity of CLEO was 100% at the concentration of 100 μL/mL. In the same way, CLEO destroyed 27, 53.3, 100 and 100% of the protoscoleces at the dose of 50 μL/mL after 5, 10, 20 and 30 min of application, respectively. The results showed that with increasing treatment time to CLEO, the percentage of mortality was remarkably raised (*P* < 0.05). The percentage of mortality also in the negative and positive controls was 4.3% after 30 min and 100% after 5 min of treatment, respectively.

**Table 2:** In vitro protoscolicidal effects of *C. longa* essential oil against protoscoleces of hydatid cyst at various concentrations following various exposure times

Concentration (μL/mL(	Exposure time (min)	Mean of mortality rate (%)
200	5	100 ± 0.0
	10	–
	20	–
	30	–
100	5	76.6 ± 3.6
	10	100 ± 0.0
	20	–
	30	–
50	5	27.0 ± 3.15
	10	53.3 ± 2.88
	20	100 ± 0.0
	30	–
Normal saline + tween 20	5	0.66 ± 0.57
	10	2.6 ± 1.15
	20	3.0 ± 0.5
	30	4.3 ± 1.15
Ag-nitrate	5	71.6 ± 2.88
	10	100 ± 0.0
	20	–
	30	–

### Ex vivo protoscolicidal activity

**Fig. 2:**
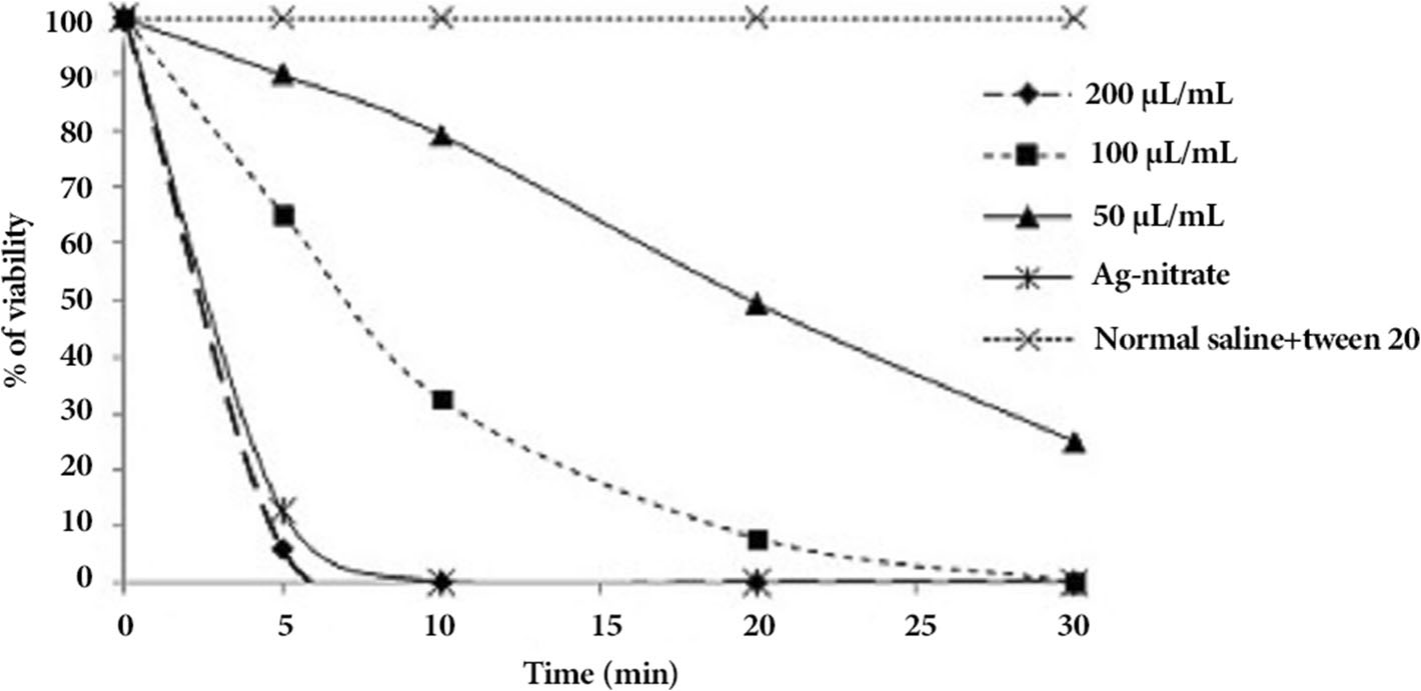
Ex vivo protoscolicidal effects of *C. longa* essential oil against protoscoleces of hydatid cyst at various concentrations following various exposure times

Figure 2 exhibited the ex vivo protoscolicidal activity of CLEO at the doses of 50, 100, and 200 μL/mL against hydatid protoscoleces. Although CLEO at the doses of 50, and 100 μL/mL showed potent effects in vitro; but did not show the similar effect in the ex vivo analysis, needing a more time to approve a notable protoscolicidal activity. Nevertheless, at the dose of 200 μL/mL and an exposure time of 5 min, approximately killed all the protoscoleces inside the cyst.

### Acute toxicity

According to the results, the LD_50_ value of intraperitoneal inoculation of the CLEO was 1.93 mL/kg of bw and the maximum nonfatal dose was1.16 mL/kg of body weight.

### Clinical chemistry and hematological parameters

The doses of 0.15, 0.3 and 0.6 mL/kg of CLEO were chosen according to the obtained LD_50_. The results showed that no death was observed after 14 days of intraperitoneal administration of *C. longa* essential oil in doses of 0.15, 0.3 and 0.6 mL/kg. As shown in Tables 3 and 4, there was no significant difference (*P* > 0.05) between biochemical and hematological factors after the oral administrations of CLEO at the aforementioned doses and control.

**Table 3:** Clinical chemistry parameters in mice sera following oral administration of *C. longa* essential oil for 2 weeks

Parameters	*C. longa* essential (mL/kg)			Control
	0.15	0.3	0.6	
AST (U/L)	142.3 ± 11.5	131 ± 9.3	137 ± 12.5	141 ± 13.5
ALT (U/L)	88.6 ± 3.3	97.3 ± 4.6	104.5 ± 7.6	93.4 ± 8.3
ALP (U/L)	248.3 ± 23.3	239.6 ± 19.5	251.5 ± 20.4	235.6 ± 11.5
Cr (mg/dL)	0.4 ± 0.05	0.37 ± 0.08	0.46 ± 0.1	0.35 ± 0.05
BUN (mg/dL)	43.3 ± 7.2	29.6 ± 8.3	39.3 ± 7.1	34 ± 3.4
TB (mg/dL)	0.79 ± 0.11	0.69 ± 0.15	0.81 ± 0.2	0.76 ± 0.2
DB (mg/dL)	0.38 ± 0.06	0.28 ± 0.03	0.39 ± 0.05	0.33 ± 0.01

*AST* aspartate aminotransferase, *ALT* alanine aminotransferase, *ALP* alkaline phosphatase, *Cr* creatinine, *BUN* Blood urea nitrogen, *TB* Total bilirubin, *DB* Direct bilirubin

**Table 4:** Hematology parameters in whole blood of mice following oral administration of *C. longa* essential oil for 2 weeks

Parameters	*C. longa* essential (mL/kg)			Control
	0.15	0.3	0.6	
RBC (×l0^6^/μL)	3.8 ± 0.15	3.2 ± 0.25	4.2 ± 0.41	3.4 ± 0.3
HGB (g/dL)	11.6 ± 0.7	10.7 ± 1.17	12.3 ± 0.6	11.3 ± 0.45
Hct (%)	31.6 ± 3.1	35.1 ± 2.51	35.2 ± 2.6	32.6 ± 2.18
WBC (×l0^3^/μL)	3.3 ± 0.35	2.6 ± 0.16	3.4 ± 0.25	2.9 ± 0.2
PLT (×l0^3^/μL)	177 ± 15	212 ± 18	197 ± 12	185 ± 17

*RBC* red blood cell, *HGB* hemoglobin, *Hct* hematocrit, *WBC* white blood cell, *PLT* platelet

## Discussion

Historically, herbal medicines have been a popular form of complementary and alternative medicine worldwide [9]. Based on the World Health Organization (WHO) reports, in excess of 70% of the world’s people trust in folk remedy for their some health care requirements.

Since protoscolicidal agents used during hydatid cyst surgery have side effects such as sclerosing cholangitis, so more attention is paid to the toxicity of these drugs, as well as the search for a suitable alternative drug [2]. This is the first known study to evaluate the efficacy of *C. longa* essential oil against hydatid protoscoleces and also its safety in the mice. Results demonstrated that *C. longa* essential oil killed 100% of protoscoleces at the doses of 200 and 100 μL/mL after 5 and 10 min of treatment, respectively.

In this study, we established the PAIR technique, with some changes, by hydatid cysts collected from sheep livers. The scolicidal agent should fill the complete cyst cavity, seeking interaction with the protoscoleces, which occasionally are placed in the cyst. Ex vivo assay displayed that although CLEO at the doses of 50, and 100 μL/mL showed potent effects in vitro; but did not show the similar effect in the ex vivo analysis, needing a more time to approve a notable protoscolicidal activity. While, *C. longa* essential oil at the dose of 200 μL/mL and an treatment time of 5 min, approximately killed all the protoscoleces inside the cyst. These findings discovered that the scolicidal activity of *C. longa* essential oil is as good as to the current scolicidal agents such as 20% hypertonic saline, and silver nitrate, etc.

The previous study confirmed that an appropriate protoscolicidal agent is characterized by its capability at lesser doses, high efficiency in a lower time, and steadiness in the cystic contents, high accessibility, minor toxicity, and capacity for fast preparation [11]. The findings suggested that *C. long* might be a natural resource for producing a novel protoscolicidal drug which can used in hydatid cyst surgery.

By GC/MS analysis, The key constituents were α-turmerone (27.1%), β- turmerone (21.8%), l-phellandrene (8.8%), and ρ-cymene (5.4%), respectively. Similarly, the essential oil of *C. longa* rhizome has been studied in detail in a number of studies [15, 16]. and the main constituents were found to be ar-turmerone and turmerol. However, the previous studies have demonstrated that the chemical composition of EO rely on species, typical weather, harvest time, and growth step, which could change the biological characteristics of plants [17].

Turmerone and ar-turmerone are, according to their chemical structure, oxygenated sesquiterpenes of the bisabolene type, responsible for turmeric’s aroma and smell [24]. Regarding the antimicrobial mechanism of some terpenoid constituents, researchers have demonstrated that these constituents exhibit their antimicrobial mechanisms through penetration into the microbe and extinction of its cell wall [25]. On the other hand, some studies have shown that these contituents, after entering the pathogens, can display their antimicrobial mechanisms by breaking down important and vital intracellular reactions and activities [26, 27].

The obtained results revealed that the LD_50_ value of the *C. longa* essential oil was 1.93 mL/kg/bw, and the maximum nonfatal dose was 1.16 mL/kg/bw. In the present study, we examined clinical and hematological parameters in the treatment of mice receiving essential oil for 14 days to assess the sub-acute toxicity of *C. longa* essential oil. Liver and renal enzyme activities such as ALT, AST, ALP, Bilirubin (total, direct), Cr, and BUN are the most important characteristics of liver and renal function. Here, we observed no considerable difference (*P* > 0.05) in the clinical chemistry and hematological factors following intraperitoneal administrations of *C. longa* essential at the doses of 0.15, 0.3, and 0.6 mL/kg for 2 weeks. Therefore, based on the standard classification of toxicity, *C. longa* essential oil did not show any considerable toxicity against NIH mice [28].

## Conclusion

The findings demonstrated that *C. longa* revealed the promising scolicidal effects against hydatid cyst protoscoleces in vitro and ex vivo. Nevertheless, additional studies will be desired to prove these outcomes by examination the essential oil as a new scolicidal agent in a clinical setting.

## Data Availability

All data generated or analyzed during this study are included in this published article.

## Abbreviations

ALP: Alkaline phosphatase
ALT: Alanine aminotransferase
AST: Aspartate aminotransferase
BUN: Blood urea nitrogen
CE: Cystic echinococcosis
CLEO: *Curcuma longa* essential oil
Cr: Creatinine
EDTA: Ethylenediaminetetraacetic acid (EDTA) anticoagulant
GC/MS: Gas chromatography/mass spectrometry
Hb: Hemoglobin
Hct: Hematocrit
Plt: Platelet
RBC: Red blood cell
WBC: White blood cell counts
